# Correlation Between Objective Habit Metrics and Objective Medication Adherence: Retrospective Study of 15,818 Participants From Clinical Studies

**DOI:** 10.2196/63987

**Published:** 2025-02-06

**Authors:** Antoine Pironet, L Alison Phillips, Bernard Vrijens

**Affiliations:** 1 AARDEX Group Seraing Belgium; 2 Iowa State University Ames, IA United States

**Keywords:** medication adherence, compliance, habit, history, correlation, association, intake, electronic database, retrospective, medication, drug, adherence

## Abstract

**Background:**

Medication adherence, or how patients take their medication as prescribed, is suboptimal worldwide. Improving medication-taking habit might be an effective way to improve medication adherence. However, habit is difficult to quantify, and conventional habit metrics are self-reported, with recognized limitations. Recently, several objective habit metrics have been proposed, based on objective medication-taking data.

**Objective:**

We aim to explore the correlation between objective habit metrics and objective medication adherence on a large dataset.

**Methods:**

The Medication Event Monitoring System Adherence Knowledge Center, a database of anonymized electronic medication intake data from ambulant participants enrolled in past clinical studies, was used as the data source. Electronic medication intake data from participants following a once-daily regimen and monitored for 14 days or more were used. Further, two objective habit metrics were computed from each participant’s medication intake history: (1) SD of the hour of intake, representing daily variability in the timing of medication intakes, and (2) weekly cross-correlation, representing weekly consistency in the timing of medication intakes. The implementation component of medication adherence was quantified using (1) the proportion of doses taken and (2) the proportion of correct days.

**Results:**

A total of 15,818 participants met the criteria. These participants took part in 108 clinical studies mainly focused on treatments for hypertension (n=4737, 30%) and osteoporosis (n=3353, 21%). The SD of the hour of intake was significantly negatively correlated with the 2 objective adherence metrics: proportion of correct days (Spearman correlation coefficient, *ρ*_S_=–0.62, *P*<.001) and proportion of doses taken (*ρ*_S_=–0.09, *P*<.001). The weekly cross-correlation was significantly positively correlated with the 2 objective adherence metrics: proportion of correct days (*ρ*_S_=0.55, *P*<.001) and proportion of doses taken (*ρ*_S_=0.32, *P*<.001). A lower daily or weekly variability in the timing of medication intakes is thus associated with better medication adherence. However, no variability is not the norm, as only 3.6% of participants have 95% of their intakes in a 1-hour window. Among the numerous factors influencing medication adherence, habit strength is an important one as it explains over 30% of the variance in medication adherence.

**Conclusions:**

Objective habit metrics are correlated to objective medication adherence. Such objective habit metrics can be used to monitor patients and identify those who may benefit from habit-building support.

## Introduction

Medication adherence is “the process by which patients take their medication as prescribed” [1]. It comprises 3 components: initiation of the treatment, correct implementation of the prescribed regimen, and persistence to the treatment [1]. Poor medication adherence is a global public health issue [2,3] and has important negative consequences on the personal level [3-6], but also at the societal level [3-5]. Interventions aiming at improving medication adherence are abundant in the literature, but few have been shown to be effective across a population [5,6]. Most medication adherence interventions have focused on structural factors outside of the individual (simplification of the regimen, refill reminders, etc) or on behavior change interventions that target reflective or deliberative factors, such as patient education [7]. As at least half of our daily behaviors are nonreflective, but rather habitual [8], interventions that target these habitual processes may be more successful than education or persuasion-based interventions.

Habits are defined as automatic behaviors responding to recurring environmental cues [9]. As stronger medication-taking habit has been shown to be associated with better medication adherence [4,10-13], in particular its implementation component, some successful interventions have focused on improving habit [5,14]. However, medication-taking habit, and habit in general, is difficult to quantify [8]. Conventional habit metrics are self-reported [12], with an important example being the Self-Reported Habit Index [15], a 12-item questionnaire. Another example is the Self-Reported Behavioral Automaticity Index [16], a 4-item subset of the Self-Reported Habit Index. These and other self-reported indices endure common limitations associated with self-reported metrics, such as social desirability bias and poor patient recall [13].

Medication-taking habit can also be assessed using objective medication-taking data [17]. Such data is collected using smart medication packages, which can take several forms: an electronic cap fitted on a medication bottle [17], an inhaler with a chip embedded [13], a blister that detects when a pill is expressed out of a cavity, etc. The common feature of smart medication packages is that they passively timestamp each time a patient accesses their medication, thus providing objective data on when a patient takes their medication. This detailed information can be used to derive habit metrics quantifying the consistency of medication intake behavior over time [11,17]—the validity of which rests on the fact that habits are context-stable responses to conditioned cues. Such habit metrics are objective and do not endure the limitations associated with self-reported habit.

Day-to-day consistency of the timing of medication intake is frequently used as an objective habit metric. It has been operationalized as the variance of the hour of intake [17], its SD [13,18], or the proportion of medication intakes occurring in a fixed-size window, for instance, 2 [4,19,20], 3 [21,22], or 4 hours [23].

These day-to-day consistency measures will penalize someone for having different routines on different days of the week (for instance, systematically taking their medication at 6 PM on weekdays and 10 PM on weekends). However, this feature might not be desirable if the cue for medication-taking (eg, breakfast) is the same throughout the week, reflecting a good medication-taking habit. To overcome this limitation, Phillips et al [11] recently introduced another metric, called the weekly cross-correlation, based on the weekly consistency of intake timing. In this metric, the medication intake timing of each day of a given week is compared to the corresponding day of the next week.

Recently also, Hoo et al [13] introduced a “pragmatic habit index” empirically defined as the product of 2 variables: stability, measured as the SD of the hour of intake and frequency, measured as the proportion of prescribed doses taken. Introducing behavioral frequency in a habit index has been criticized [16], as it incorporates the dependent variable of interest (behavior frequency) in the predictors.

In a recent study on 79 patients with type 2 diabetes, objective habit metrics were found to correlate with objective medication adherence [11]. The goal of this paper is to reinvestigate this correlation on a 200-fold larger dataset covering multiple pathologies, introduced in the next section.

The main hypothesis is that a more consistent medication-taking habit is correlated to better medication adherence.

## Methods

### Data

This study was a retrospective cohort study. AARDEX Group’s database, the Medication Event Monitoring System (MEMS; AARDEX Group) Adherence Knowledge Center was used as the data source. This database contains anonymized electronic medication adherence data from ambulant participants enrolled in clinical studies that ran between 1989 and 2016. These participants’ medication adherence was electronically monitored using the MEMS. The following selection criteria were used: (1) once-daily medication with (2) a follow-up longer than 14 days. This second criterion was required because 14 days is the minimum duration needed to compute the weekly cross-correlation. These selection criteria matched data from 15,818 participants totaling 3,053,779 medication intakes.

### Ethical Considerations

#### Human Participants’ Ethics Review Approval or Exemptions

Approval from an institutional review board was not required for the present analysis, as it consists of secondary research for which the identity of the participants is unknown, per Title 45 of the Code of Federal Regulations Part 46, Subpart A, Section 46.104, Paragraph (d)(4)(ii) [24]. The original data collection for all studies included in this analysis was approved by institutional review boards.

#### Informed Consent

Data were obtained from participants enrolled in clinical studies. As such, the participants provided informed consent for the procedures of the original studies. These procedures required participants to store their medication in electronic medication packages. Participants were informed that these electronic medication packages recorded their medication intakes. The original study procedures also included using the generated data to monitor the participants’ dosing history and analyze their medication intake behavior. As a consequence, informed consent was not sought for this secondary analysis, because it corresponds to the use of the data that was originally presented to participants.

#### Privacy and Confidentiality

The data in AARDEX Group’s database, the Adherence Knowledge Center, is anonymized.

#### Compensation Details:

Participants were compensated for their participation in the original clinical studies. Compensation modalities varied between studies and countries, and the authors do not possess information about the compensation process, which was managed by the sponsors of the original studies.

### Objective Habit Metrics

For each participant, 2 objective habit metrics were computed. The first objective habit metric is the SD of the day-to-day hour of intake. The hours and minutes are extracted for each medication intake timestamp, irrespective of the date of intake, and the SD of the resulting list of hours and minutes is computed. The lower the SD, the more consistent a person is in the timing of their medication intakes. In the extreme case, when SD equals 0 h, all intakes occur at exactly the same time of the day.

The second objective habit metric is the “weekly cross-correlation” introduced by Phillips et al [11]. This metric compares each day of a given week with the corresponding day of the next week and quantifies whether intakes occurred around the same time on these 2 days. To do so, medication intakes are represented in a 2D matrix, denoted *A*, of dimensions 24 × *N_days_*, with *N_days_* being the number of follow-up days. The matrix is initially filled with zeros. Each medication intake occurring on day *l* at hour *k* is translated as a value of 1 for element *A*[*k*,*l*]. Then, each element *A*[*k*,*l*] of the matrix is multiplied by its matching element from the previous week, A[*k*,*l – 7*]. If intakes occur at the same time from week to week, the product will be close to 1. Otherwise, it will be close to zero. Finally, the weekly cross-correlation is equal to the sum of all element-wise products divided by the Euclidean norm of *A*. More details and code explaining how to compute these metrics are presented in Multimedia Appendix 1. As the pragmatic habit index incorporates adherence, it was not included in the present analysis.

### Implementation Adherence Metrics

Medication adherence, more precisely its implementation component [1], was quantified as the proportion of correct days, that is, the proportion of days with exactly 1 intake. A second implementation metric was used: the proportion of doses taken. The proportion of doses taken was computed as the ratio between the total number of doses taken and the prescribed number of doses. In this work, since participants were on a once-daily regimen, the prescribed number of doses was equal to the number of follow-up days. If a participant discontinued treatment too early, the analysis was limited to the period during which the participant was on treatment.

The hypotheses were that (1) SD of the day-to-day hour of intake would be negatively correlated to objective adherence (the more variable the hour of intake, the worse the adherence); (2) the weekly cross-correlation would be positively correlated to objective adherence (the more consistent the pattern of intakes, the better the adherence); and (3) the weekly cross-correlation would be more strongly correlated to objective adherence than the SD of the hour of intake.

All dosing history data were compiled while patients were engaged with the monitored medication in the trial. Therefore, noninitiation and nonpersistence are not part of the adherence evaluation in this research.

### Statistical Analyses

Continuous variables were reported using medians and first and third quartiles. Categorical variables were reported using counts and proportions. Pairwise correlations between continuous variables were assessed using the Spearman rank correlation coefficient. Correlation coefficients were converted using Fisher *Z*-transformation to obtain CIs [25,26]. Correlation coefficients were compared by performing a *z*-test on the difference between their Fisher *Z*-transformations [26,27]. The proportion of variance in adherence explained by habit was quantified using the Pearson correlation coefficient, squared [26]. Analyses were performed using Python 3 (Python Software Foundation) [28].

## Results

The 15,818 participants whose data was extracted from the MEMS Adherence Knowledge Center took part in 108 clinical studies. These studies enrolled a median of 59 (IQR 22-169) participants. Table 1 presents the characteristics of the population.

**Figure 1:**
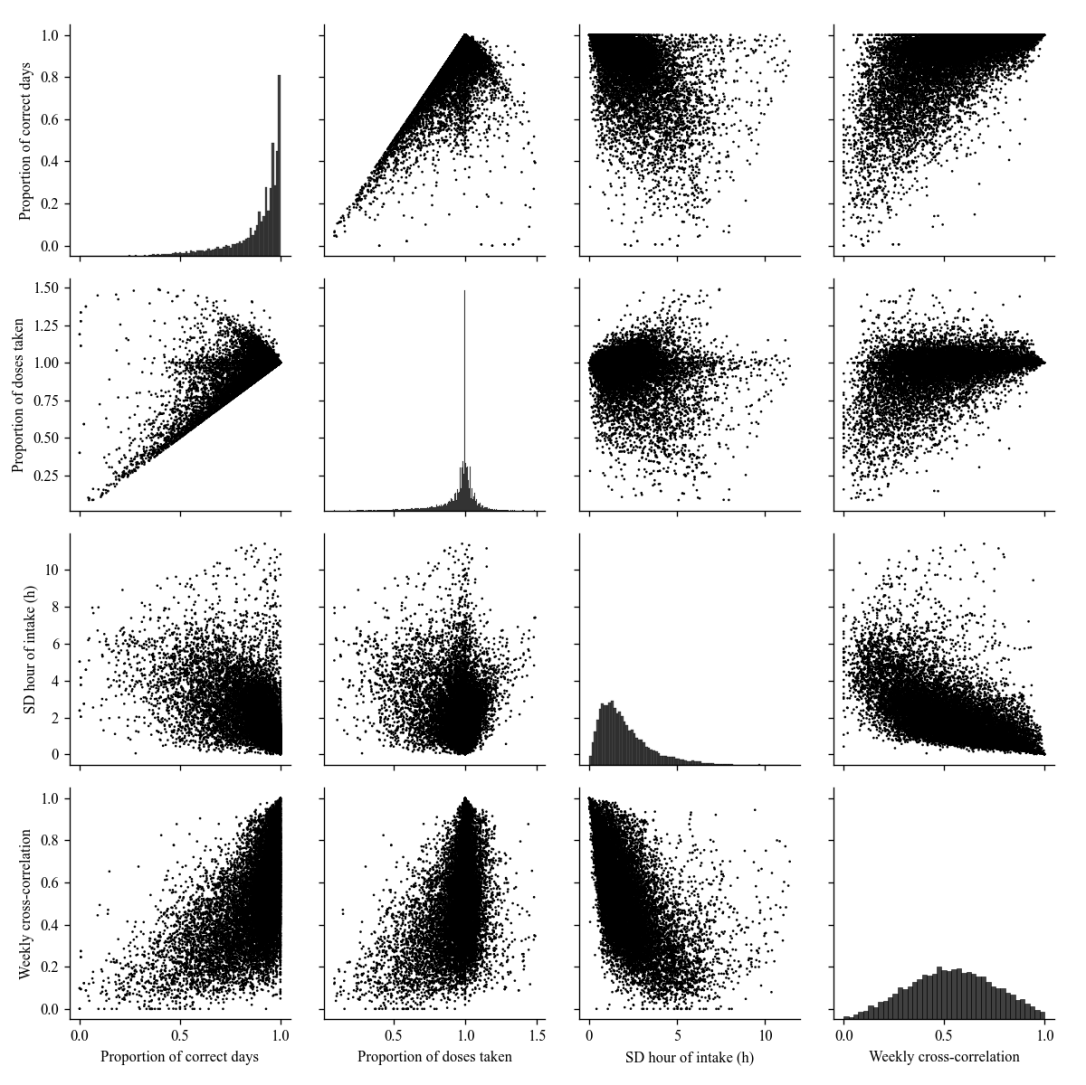
Pairwise relationships between the four variables of interest: 2 objective adherence metrics: proportion of correct days (number 1) and proportion of doses taken (number 2) and 2 objective habit metrics (numbers 3 and 4). Off-diagonal panels contain scatter plots for each pair of variables; on-diagonal panels show a histogram of the distribution of single variables.

Figure 1 presents the distributions and pairwise plots for the 4 variables of interest: proportion of correct days and proportion of doses taken as 2 objective measures of adherence, and SD of the hour of intake and weekly cross-correlation as 2 objective habit metrics. According to the 2 top-left diagonal panels, the proportion of correct days ranges between 0 and 1 by definition, while the proportion of doses taken ranges between 0 and 1.5 indicating that some participants took more doses than prescribed. The scatter plots also show that the proportion of doses taken is correlated to the proportion of correct days. In addition, the first is always superior to the second, which is a consequence of their definition.

According to the 2 bottom-right diagonal panels of Figure 1, the SD of the hour of intake ranges between 0 and 12 hours, while the weekly cross-correlation ranges between 0 and 1. These two findings are direct consequences of the definitions of these variables. According to the scatter plots, these 2 variables are negatively correlated, indicating that a larger consistency in day-to-day timing (low SD hour of intake) is associated with a larger consistency in week-to-week timing (high weekly cross-correlation).

Table 2 presents the associated correlation coefficients. All 4 Spearman correlation coefficients were significantly different from zero (T scores for the significance of the coefficients, from left to right and top to bottom: –61.48, –10.70, 104.11, and 48.59 with a df of 15,816, all *P*<.001).

According to Figure 1 and Table 2, the correlation between the SD of the hour of intake and objective adherence (measured using the proportion of correct days or proportion of doses taken) has a negative sign, indicating that, as hypothesized, the more day-to-day consistency in timing the higher the adherence. Figure 1 and Table 2 also show that the weekly cross-correlation is positively correlated to objective adherence, meaning that the more consistent a person’s medication intake pattern is from week to week, the higher their medication adherence, which is also in line with the hypotheses.

The comparison of the Spearman correlation coefficients presented in Table 2 shows that the SD of the hour of intake is more strongly correlated to the proportion of correct days than the weekly cross-correlation is (95% CI for the difference between absolute values of Spearman coefficients: 0.05 to 0.09, *z* score for this difference=21.46, *P*<.001), conversely to what was hypothesized. On the other hand, the weekly cross-correlation is more strongly correlated to the proportion of doses taken than the SD hour of intake is (CI for the difference between absolute values of Spearman coefficients: 0.25 to 0.21, *z* score for this difference=9.45, *P*<.001), as hypothesized.

Habit accounts for about 30% of the variance in medication adherence, measured using the proportion of correct days: 29.18% for SD of the hour of intake (95% CI 29.17% to 29.19%) and 31.25% for the weekly cross-correlation (95% CI 31.24% to 31.26%). When medication adherence is measured using the proportion of doses taken, the proportion of variance explained by habit is smaller: 4.91% for the SD of the hour of intake (95% CI 4.91% to 4.92%) and 15.74% for the weekly cross-correlation (95% CI 15.73% to 15.75%).

**Table 1:** Population characteristics.

Pathology	Values
Hypertension, n (%)	4737 (30)
Osteoporosis, n (%)	3353 (21)
Viral hepatitis, n (%)	2005 (13)
Hypercholesterolemia, n (%)	1246 (8)
Angina, n (%)	968 (6)
AIDS, n (%)	754 (5)
Depression, n (%)	561 (4)
Diabetes, n (%)	549 (3)
Reversible airway obstruction, n (%)	397 (3)
Heart failure, n (%)	334 (2)
Attention-deficit/hyperactivity disorder, n (%)	301 (2)
Colorectal polyp, n (%)	192 (1)
Others, n (%)	421 (3)
Follow-up duration (days), median (IQR)	168 (60-365)
Proportion of correct days (%), median (IQR)	93.3 (83.7-97.7)
Proportion of doses taken (%), median (IQR)	100 (93.7-101.8)
SD hour of intake (h), median (IQR)	1.7 (1-2.7)
Weekly cross-correlation, median (IQR)	0.54 (0.38-0.69)

**Table 2:** Spearman correlation coefficients with 95% CI between 2 objective habit metrics (rows) and 2 objective adherence metrics (columns).

Objective habit	Objective adherence	
	Proportion of correct days	Proportion of doses taken
SD hour of intake	–0.62 (–0.63 to –0.61)	–0.09 (–0.1 to –0.07)
Weekly cross-correlation	0.55 (0.54 to 0.56)	0.32 (0.3 to 0.33)

## Discussion

### Principal Findings

This work showed that medication-taking habit is positively correlated to medication adherence in a very large, cross-pathology population of participants enrolled in clinical studies. Medication-taking habit was quantified using 2 measures of the consistency in the timing of medication intakes: day-to-day and week-to-week. The implementation component of medication adherence was quantified using 2 measures: the proportion of correct days and the proportion of doses taken. The conclusion was the same irrespective of the habit or adherence measure: the stronger the habit, the better the medication adherence.

The results showed no clear superiority of any of the 2 objective habit metrics per the strength of their correlation with adherence. The correlation of objective habit metrics with self-reported habit metrics, which are the most frequently used habit metrics, might be used to differentiate the 2 objective habit metrics. Previous studies exploring this correlation are discussed in the next section.

### Comparison to Prior Work

The link between objective habit metrics and medication adherence has been investigated in very few other studies. The first one [11], performed by the authors on a much smaller dataset, reported very similar results: (1) a negative correlation between a measure of day-to-day variability and the proportion of correct days and (2) a positive correlation between the weekly cross-correlation and the proportion of correct days. The second one [13], previously discussed, used a habit metric incorporating adherence and reported an expected correlation between these 2 quantities.

Over 700 determinants of adherence have been reported [29]. Among all these determinants, objective habit strength seems to be an important one, as it explained over 30% of the variance in medication adherence in this work. This observation empirically justifies studying objective medication-taking habit.

A question related to the present work is whether people use time-based cues for taking their medications [10]. To answer this question, the proportion of participants having 95% of their intakes in a 1-hour window was computed. Only 3.6% of participants met this criterion. In a previous study [10], 21.7% of participants reported taking their medication at a specific time of the day. These numbers mean that a minority of patients rely on cues triggered by the clock and take medication at the same time every day. This finding might explain why reminders for medication adherence sent at a specific time of the day can be ineffective [30].

Few studies have studied how well objective habit metrics for medication adherence correlate with conventional self-reported habit metrics. SD or other metrics of day-to-day consistency were found to correlate with self-reported habit in 2 studies [17,18], but not in a third one [11]. Weekly cross-correlation was found to correlate with self-reported habits in 1 study [11].

### Strengths and Limitations

First, the 2 objective habit metrics used in this study are measures of consistency in timing, day-to-day, or week-to-week. However, not all habits translate into consistency in timing, and consistency in timing can originate from other factors than habit [9]. In such cases, the objective habit metrics will not reliably quantify the presence or absence of habit. On the other hand, the objective consistency metrics have the advantage that they do not endure biases associated with self-reported habit metrics, such as recall bias.

Second, the dataset used in this paper was very large, so the findings are likely to be generalizable to the whole population of participants enrolled in clinical trials. On the other hand, no conclusion about a specific disease, population, or time can be drawn, because there would be a large confounding influence of the underlying study design and specific medication characteristics. The main result is that, in general, adherence is related to objective habit.

Third, in this study, habit and medication adherence were computed over a participant’s whole follow-up, until treatment discontinuation. However, for a single participant, habit strength may have changed throughout the duration of this study, for instance, because of the introduction of a new device for medication adherence monitoring. The evidence on the effects that electronic adherence monitoring causes on adherence itself is unclear [22,31]. The effects of electronic adherence monitoring on medication-taking habit have never been studied, to the best of the authors’ knowledge.

Another reason that may have caused individual changes in habit strength is that some of the studies analyzed were testing habit-building interventions. In such settings, the objective habit metrics used in this paper can be computed over shorter periods to capture the dynamics of habit formation and maintenance. For instance, in the case of a specific habit-building intervention, Pironet et al [32] observed that habit strength increased after a 6-month intervention and remained stable 6 months after the intervention was stopped.

### Future Directions

If a patient’s implementation adherence is measured electronically, their habit can be computed continuously from the recorded data and serve as a support for an intervention, in-person or through a mobile app. The intervention could focus on providing support to build a better medication-taking habit. An example of such a mobile intervention is presented in the study by Stawarz et al [33].

This work investigated the relationship between habit and the implementation component of medication adherence. Other, evenly important questions are whether objective habit indices predict the quality of implementation over the longer term, and if objective habit indices predict early treatment discontinuation.

### Conclusion

In this study, the correlation between objective habit metrics, computed from electronic medication intake data, and objective medication adherence was assessed in a large database of participants enrolled in past clinical trials. The 2 objective habit metrics tested in this work were correlated to the 2 objective adherence metrics. These 4 pairwise correlations all imply that a lower variability in the pattern of medication intakes, be it day-to-day or week-to-week, is correlated with higher medication adherence. However, no variability is not the norm.

Objective habit metrics allow us to better identify patients who might benefit from habit-building support. If a patient uses electronic monitoring, the habit metrics can even be automated and computed in real time, directly reflecting habit changes and allowing for timely intervention. In addition, some effective interventions for medication adherence are based on developing or improving habits [5]. In such settings, electronic monitoring can be used to assess the effect of the habit-building intervention in real time [32].

## Appendix

Multimedia Appendix 1 Objective habit strength metrics.

## Abbreviations

MEMS: Medication Event Monitoring System

## Data Availability

The individual values of objective medication adherence (proportion of correct days and proportion of doses taken) and objective habit (SD of the hour of intake and weekly cross-correlation) can be downloaded on AARDEX Group’s website, in the section “Knowledge Center.”

## References

[1] Vrijens B, De Geest S, Hughes DA, Przemyslaw K, Demonceau J, Ruppar T, Dobbels F, Fargher E, Morrison V, Lewek P, Matyjaszczyk M, Mshelia C, Clyne W, Aronson JK, Urquhart J, ABC Project Team (2012). A new taxonomy for describing and defining adherence to medications. Br J Clin Pharmacol.

[2] Khan R, Socha-Dietrich K (2018). Investing in medication adherence improves health outcomes and health system efficiency: adherence to medicines for diabetes, hypertension, and hyperlipidaemia. OECD Health Working Papers.

[3] Jimmy B, Jose J (2011). Patient medication adherence: measures in daily practice. Oman Med J.

[4] Phillips LA, Leventhal H, Leventhal EA (2013). Assessing theoretical predictors of long-term medication adherence: patients' treatment-related beliefs, experiential feedback and habit development. Psychol Health.

[5] Russell CL, Hathaway D, Remy LM, Aholt D, Clark D, Miller C, Ashbaugh C, Wakefield M, Ye S, Staggs VS, Ellis RJ, Goggin K (2020). Improving medication adherence and outcomes in adult kidney transplant patients using a personal systems approach: SystemCHANGE™ results of the MAGIC randomized clinical trial. Am J Transplant.

[6] Costa E, Giardini A, Savin M, Menditto E, Lehane E, Laosa O, Pecorelli S, Monaco A, Marengoni A (2015). Interventional tools to improve medication adherence: review of literature. Patient Prefer Adherence.

[7] Kini V, Ho PM (2018). Interventions to improve medication adherence: a review. JAMA.

[8] Wood W, Quinn JM, Kashy DA (2002). Habits in everyday life: thought, emotion, and action. J Pers Soc Psychol.

[9] Volpp KG, Loewenstein G (2020). What is a habit? Diverse mechanisms that can produce sustained behavior change. Organ Behav Hum Decis Process.

[10] Rajpura JR (2014). Capsule commentary on Brooks et al., strategies used by older adults with asthma for adherence to inhaled corticosteroids. J Gen Intern Med.

[11] Phillips LA, Pironet A, Vrijens B (2023). Evaluating objective metrics of habit strength for taking medications. J Behav Med.

[12] Badawy SM, Shah R, Beg U, Heneghan MB (2020). Habit strength, medication adherence, and habit-based mobile health interventions across chronic medical conditions: systematic review. J Med Internet Res.

[13] Hoo ZH, Wildman MJ, Campbell MJ, Walters SJ, Gardner B (2019). A pragmatic behavior-based habit index for adherence to nebulized treatments among adults with cystic fibrosis. Patient Prefer Adherence.

[14] Conn VS, Ruppar TM (2017). Medication adherence outcomes of 771 intervention trials: systematic review and meta-analysis. Prev Med.

[15] Verplanken B, Orbell S (2003). Reflections on past behavior: a self-report index of habit strength. J Appl Soc Pyschol.

[16] Gardner B, Abraham C, Lally P, de Bruijn G (2012). Towards parsimony in habit measurement: testing the convergent and predictive validity of an automaticity subscale of the Self-Report Habit Index. Int J Behav Nutr Phys Act.

[17] Phillips LA, Burns E, Leventhal H (2021). Time-of-day differences in treatment-related habit strength and adherence. Ann Behav Med.

[18] van de Vijver I, Brinkhof LP, de Wit S (2023). Age differences in routine formation: the role of automatization, motivation, and executive functions. Front Psychol.

[19] Linnemayr S, Odiit M, Mukasa B, Ghai I, Stecher C (2024). Incentives and reminders to improve long-term medication adherence (INMIND): impact of a pilot randomized controlled trial in a large HIV clinic in Uganda. J Int AIDS Soc.

[20] Phillips LA, Cohen J, Burns E, Abrams J, Renninger S (2016). Self-management of chronic illness: the role of 'habit' versus reflective factors in exercise and medication adherence. J Behav Med.

[21] Russell CL, Conn VS, Ashbaugh C, Madsen R, Hayes K, Ross G (2006). Medication adherence patterns in adult renal transplant recipients. Res Nurs Health.

[22] McGrady ME, Ramsey RR (2020). Using electronic monitoring devices to assess medication adherence: a research methods framework. J Gen Intern Med.

[23] Stecher C, Mukasa B, Linnemayr S (2021). Uncovering a behavioral strategy for establishing new habits: evidence from incentives for medication adherence in Uganda. J Health Econ.

[24] (2019). Exemptions (2018 requirements). Office for Human Research Protections.

[25] Altman DG (1999). Practical Statistics for Medical Research.

[26] Cohen J, Cohen P, West S, Aiken L (2003). Applied Multiple Regression/Correlation Analysis for the Behavioral Sciences.

[27] Myers L, Sirois MJ (2006). Spearman correlation coefficients, differences between. Encyclopedia of Statistical Sciences.

[28] Van Rossum G, Drake FL (2009). Introduction to Python 3: Python Documentation Manual Part 1.

[29] Kardas P, Lewek P, Matyjaszczyk M (2013). Determinants of patient adherence: a review of systematic reviews. Front Pharmacol.

[30] Liu X, Lewis JJ, Zhang H, Lu W, Zhang S, Zheng G, Bai L, Li J, Li X, Chen H, Liu M, Chen R, Chi J, Lu J, Huan S, Cheng S, Wang L, Jiang S, Chin DP, Fielding KL (2015). Effectiveness of electronic reminders to improve medication adherence in tuberculosis patients: a cluster-randomised trial. PLoS Med.

[31] Acosta FJ, Ramallo-Fariña Y, Bosch E, Mayans T, Rodríguez CJ, Caravaca A (2013). Antipsychotic treatment dosing profile in patients with schizophrenia evaluated with electronic monitoring (MEMS®). Schizophr Res.

[32] Pironet A, Bartlett Ellis R, Stephen MB, Yerram P, Wakefield M, Awopetu D, Russell CL (2024). The SystemCHANGE intervention improves medication-taking habit.

[33] Stawarz K, Cox A, Blandford A (2014). Don't forget your pill!: designing effective medication reminder apps that support users' daily routines.

