# Supplementary material for: Correlation Between Objective Habit Metrics and Objective Medication Adherence: Retrospective Study of 15,818 Participants From Clinical Studies
Source: Interact J Med Res. 2025 Feb 6;14:e63987. doi: 10.2196/63987 (PMC11843050; doi:10.2196/63987)
Supplement: Multimedia Appendix 1 [file ijmr_v14i1e63987_app1.docx]

## SD of the hour of intake

Given the sequence of hours of intake, $h_{i}$ ($i=1,\ldots,N$), the mean hour of intake is computed as:

$$\bar{h}=\frac{1}{N}\sum_{i} h_{i}.$$

Then, the SD in hour of intake is computed as:

$$\sigma=\sqrt{\frac{1}{N}\sum_{i} \left( h_{i}-\bar{h} \right)^{2}}.$$

Using linear summary statistics (mean and SD) with a circular quantity such as hours of the day requires specifying a cutoff. If such a cutoff is not specified, it is implicitly set at midnight, meaning that medication intakes at 23:59 and at 0:01 will be numerically considered as very different, while they are not. The cutoff is usually set at 3 a.m. [1, 2], where few medication intakes are assumed to occur. From the dataset used in the present study, we determined that 0.4 % of all medication intakes occurred between 2:30 am and 3:30 am. All medication intakes occurring between midnight and 3 a.m. are thus considered as belonging to the previous day [1].

## Weekly cross-correlation

Let $t_{i}$ be the sequence of medication intakes for a given subject, with $i=1,\ldots,N$. The time of each medication intake is decomposed in days, hours and a remainder, containing minutes and seconds, as follows.

$$t_{i}=n_{days}(t_{i})\cdot24 h+n_{hours}(t_{i})\cdot60 \mathrm{minutes}+r\cdot60 \mathrm{minutes}$$

With $n_{days,i}$ a positive integer, $n_{hours,i}<24$ being a positive integer and $r<1$ being a positive real number.

Let $A$ be a null matrix with 24 rows and $N_{days}$ columns, $N_{days}$ being the number of follow-up days. $A$ is iteratively filled as follows: for each medication intake ($i=1,\ldots,N$):

If $n_{hours,i}<23$

$$A\left[ n_{hours}(t_{i}),n_{days}(t_{i}) \right]=1-r$$

$$A\left[ n_{hours}\left( t_{i} \right)+1,n_{days}(t_{i}) \right]=r$$

If $n_{hours,i}=23$,

$$A\left[ 23,n_{days}(t_{i}) \right]=1-r$$

$$A\left[ 0,n_{days}(t_{i})+1 \right]=r$$

If there are several medication intakes affecting element $A[k,l]$, their contributions are added.

Let $B[k,l]$ be a one-week shifted version of $A[k,l]$, defined as:

$$B\left[ k,l \right]=A[k,\left( l-7 \right) \mathrm{mod} N_{days}]$$

The weekly cross-correlation between $A[k,l]$ and $B[k,l]$ is:

$$\mathrm{WCC}=\frac{\sum_{k,l} A\left[ k,l \right]B\left[ k,l \right]}{\sqrt{\sum_{k,l} A\left[ k,l \right]^{2}}\sqrt{\sum_{k,l} B\left[ k,l \right]^{2}}}$$

Since the norm of $A[k,l]$ and $B[k,l]$ is the same, this expression can be further simplified as:

$$\mathrm{WCC}=\frac{\sum_{k,l} A\left[ k,l \right]B\left[ k,l \right]}{\sum_{k,l} A\left[ k,l \right]^{2}}$$

## References

1. Hoo ZH, Wildman MJ, Campbell MJ, Walters SJ, Gardner B. A pragmatic behavior-based habit index for adherence to nebulized treatments among adults with cystic fibrosis. Patient Prefer Adherence. 2019 Feb 13;13:283-294. PMID: 30863018
2. Vrijens B, Vincze G, Kristanto P, Urquhart J, Burnier M. Adherence to prescribed antihypertensive drug treatments: longitudinal study of electronically compiled dosing histories. BMJ. 2008 May 17;336(7653):1114-7. PMID: 18480115
